# Somatostatin Negatively Regulates Parasite Burden and Granulomatous Responses in Cysticercosis

**DOI:** 10.1155/2014/247182

**Published:** 2014-07-09

**Authors:** Mitra Khumbatta, Bahrom Firozgary, David John Tweardy, Joel Weinstock, Gohar Firozgary, Zal Bhatena, Tushar Bulsara, Ricardo Siller, Prema Robinson

**Affiliations:** ^1^Section of Infectious Diseases, Department of Medicine, Baylor College of Medicine, One Baylor Plaza, Houston, TX 77030, USA; ^2^Division of Gastroenterology, Department of Medicine, Tufts Medical Center, 800 Washington Street, P.O. Box 233, Boston, MA 02111, USA

## Abstract

Cysticercosis is an infection of tissues with the larval cysts of the cestode, *Taenia* *solium*. While live parasites elicit little or no inflammation, dying parasites initiate a granulomatous reaction presenting as painful muscle nodules or seizures when cysts are located in the brain. We previously showed in the *T. crassiceps* murine model of cysticercosis that substance P (SP), a neuropeptide, was detected in early granulomas and was responsible for promoting granuloma formation, while somatostatin (SOM), another neuropeptide and immunomodulatory hormone, was detected in late granulomas; SOM's contribution to granuloma formation was not examined. In the current studies, we used somatostatin knockout (SOM^−/−^) mice to examine the hypothesis that SOM downmodulates granulomatous inflammation in cysticercosis, thereby promoting parasite growth. Our results demonstrated that parasite burden was reduced 5.9-fold in SOM^−/−^ mice compared to WT mice (*P* < 0.05). This reduction in parasite burden in SOM^−/−^ mice was accompanied by a 95% increase in size of their granulomas (*P* < 0.05), which contained a 1.5-fold increase in levels of IFN-*γ* and a 26-fold decrease in levels of IL-1*β* (*P* < 0.05 for both) compared to granulomas from WT mice. Thus, SOM regulates both parasite burden and granulomatous inflammation perhaps through modulating granuloma production of IFN-*γ* and IL-1*β*.

## 1. Introduction

Cysticercosis is an infection of tissues with larval cysts of the cestode* Taenia solium*. The disease is spread mainly via the fecal-oral route and is contracted by ingestion of food and water that is contaminated with* T. solium* eggs. Following ingestion, the eggs penetrate the intestinal lumen and migrate preferentially to the muscles and brain, where they form cysts that can survive for years. While alive, cysts elicit no or minimal inflammation. On the other hand, dying parasites initiate a granulomatous reaction through pathways that are incompletely understood and manifest clinically as painful muscles nodules or seizures when the cysts are located in the brain.

Somatostatin is a neuropeptide and immunomodulatory hormone produced predominantly by macrophages that binds to receptors expressed on the surface of lymphocytes [1–5] and other cells. Somatostatin produced by macrophages within granulomatous inflammation associated with schistosomiasis downmodulates inflammatory responses in that disease [1, 6]. Treatment of* Schistosoma*-infected mice with octreotide, a somatostatin analogue, reduces granuloma size by 60% and decreases antigen-induced IFN-*γ* release by macrophages [6]. In addition, somatostatin inhibits production of TNF-*α*, IL-6, IL-10, and IFN-*γ* in other* in vivo* and* in vitro* inflammatory settings. For example, somatostatin administration reduced levels of TNF-*α* and IL-6 in the serum of patients with thyroid eye disease and in the serum of rats following lipopolysaccharide-induced septic shock [7–11]. Somatostatin also reduced levels of TNF-*α*, IL-6, and IL-1*β* produced by LPS-activated monocytes, as well as levels of IL-6, IL-10, and IFN-*γ* produced by PBMC isolated from systemic lupus erythematous patients [7–11].

Murine* Taenia crassiceps* infection of mice is widely used to model* T. solium* infection in man [12–16]. Using this model, we previously demonstrated that substance P, a neuropeptide, is produced early within granulomas elicited by dying parasites [17], while somatostatin is produced in more mature granulomas [18]. We also demonstrated that the Th1 cytokines, IFN-*γ* and IL-2, were detected in early granulomas, while Th2 cytokines, IL-4 and IL-10, were detected in more mature granulomas [19]. In addition, we showed that substance P knockout mice infected with* T. crassiceps* produced smaller granulomas than infected WT mice [17] strongly suggesting that substance P is one of the drivers of granuloma formation. The factors that downmodulate granulomatous inflammation, however, were not examined in these studies and remain ill-defined.

Granuloma formation by the host in response to chronic infectious agents is thought to be essential for limiting and eventually clearing infection. In schistosomiasis, antigens released by live eggs initiate granuloma development. As the eggs die and are absorbed, granulomas resolve leaving fibrotic plaques [20]. In cysticercosis, cysts that are dying initiate a Th1 response leading to granulomatous inflammation which help to clear the cyst. Evidence that Th1 responses help clear infection while Th2 response opposes this effect is provided by the findings that IFN-*γ* or IL-2 administration to* T. crassiceps*-infected mice results in reduced parasite numbers, while IL-10 administration mice increased parasite burden [21]. However, there are no studies that establish the correlation between extent of granulomatous inflammation,* per se*, and parasite load in cysticercosis. We hypothesized that the somatostatin is one of the factors that downmodulates granulomatous inflammation and that, by doing so, it negatively regulates parasite burden in murine cysticercosis.

To examine this hypothesis, we infected somatostatin-deficient mice with* T. crassiceps* and determined their parasite burden and granulomatous responses and compared them to the parasite burden and granulomatous responses observed in infected wild type (WT) mice. The results demonstrate that somatostatin-deficient mice had a markedly diminished parasite burden and a more robust granulomatous response. Not surprisingly, the granulomas of somatostatin-deficient mice produced higher levels of the Th1 cytokine, IFN-*γ*, compared to granulomas from WT mice. Thus, somatostatin is a negative regulator of granulomatous inflammation in* T. crassiceps* infection and its deficiency leads to decreased parasite burden.

## 2. Material and Methods

### 2.1. Mice

All studies with mice were approved by the Institutional Animal Care and Use Committee of Baylor College of Medicine (IACUC protocol no. AN209). Use of all animals involved in this project was carried out according to the provisions of the Animal Welfare Act, PHS Animal Welfare Policy, the principals of the NIH Guide for the Care and Use of Laboratory Animals, and the policies and procedures of Baylor College of Medicine. All possible steps were taken to avoid animal suffering at every stage of the experiments.

Six-week-old WT C57BL/6 mice were purchased from Jackson Laboratories. Homozygous somatostatin-deficient (SOM^−/−^) mice were the kind gift from Drs. David Elliott and Joel Weinstock, University of Iowa, and were generated, as described [22]. Briefly, a mutated* Smst* gene allele with deletion of promoter sequences and the first coding exon was generated by homologous recombination in embryonic stem cells. The somatostatin null allele contains a neo-resistance cassette, but it does not have an expressed reporter gene. Germline chimeric mice were derived by injection of C57BL/6J blastocysts with correctly targeted E14 embryonic stem cells derived from substrain 129P2/Ola mice. F_1_ heterozygous mice were obtained by mating chimeric males with C57BL/6J females. Subsequently, F_2_ (C57, 129) mice were obtained by mating F_1_ males with females, and the three expected somatostatin genotypes were obtained in normal Mendelian proportions. To reduce genetic background variability that is inherent in the original F_2_ (C57, 129) mutant strain, the somatostatin null allele was backcrossed for five successive generations onto the C57BL/6J inbred strain to produce N_5_ incipient-congenic mice. Homozygous somatostatin knockout mice were healthy and fertile and did not display physical or behavioral abnormalities.

### 2.2. Murine Cysticercosis Model

WT and SOM^−/−^ female mice were intraperitoneally infected with 10 cysts of the ORF strain of* T. crassiceps*, as described [16, 17]. Three months following infection, mice were sacrificed, their peritoneal cavity opened, and the contents (cysts and granulomas) harvested by washing the peritoneal cavity with HBSS. The washings were placed into a petridish and the cysts and granulomas were enumerated. Granulomas were flash-frozen in liquid nitrogen, weighed, and homogenized in ice-cold PBS containing protease inhibitor, aprotinin (500 KIU/mL, Sigma), followed by centrifugation at 16,000 g at 4 degrees C. Total protein in the supernatant was quantified using the Bradford method (cat no. 500-0006, Bio-Rad, Hercules, CA). IL-2, IFN-*γ*, IL-4, IL-10, IL-1*β*, IL-6, and TNF-*α* protein levels were determined by sandwich ELISA assays (R&D Systems, San Diego, California) as per the manufacturer's instructions; results are expressed as pg cytokine/mg total protein.

### 2.3. Statistical Analyses

Data presented are mean ± SEM or SD of a minimum of 2 experiments, as indicated. Statistical differences were determined using the Mann-Whitney test.

## 3. Results

### 3.1. Somatostatin Deficiency Results in Decreased Parasite Load and Increased Granuloma Size

**Figure 1:**
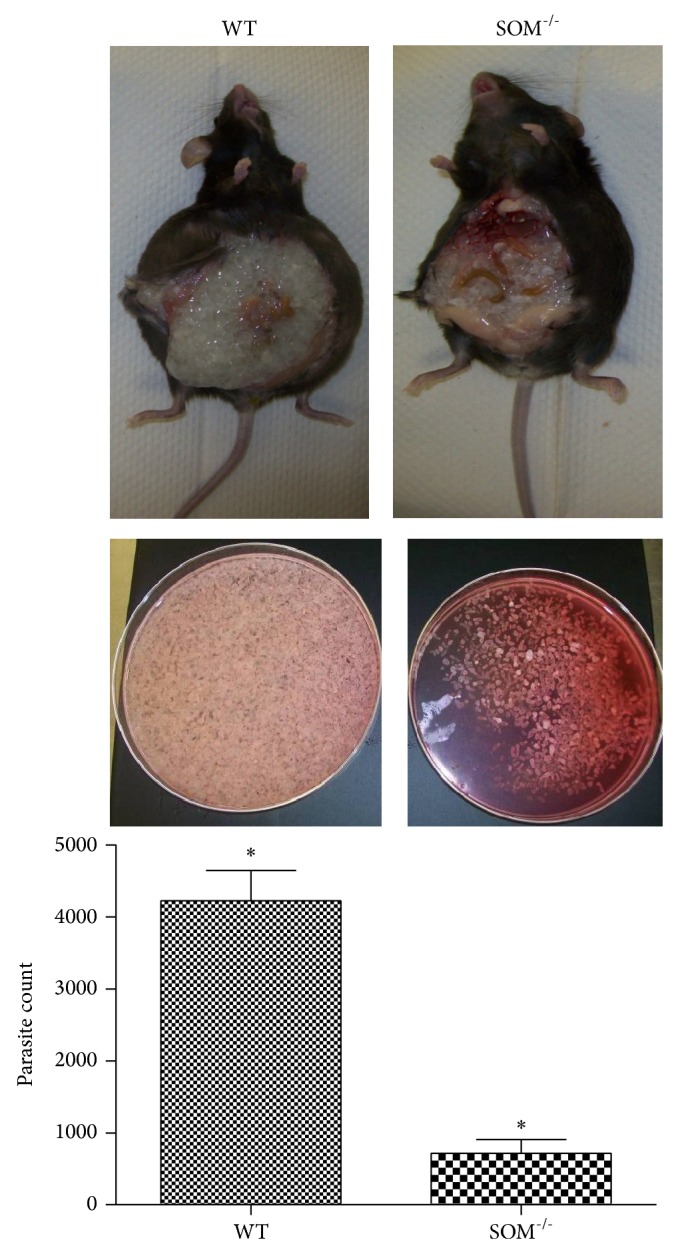
Effect of somatostatin deficiency on parasite load. Representative photographs of infected mice, WT (left side) and SOM^−/−^ (right side), are shown in the top panels following laparotomy to reveal intraperitoneal cysts. Representative photographs of cysts harvested from the peritoneal cavity are shown for each mouse in the middle panels. The bottom panel shows mean ± SD of the number of cysts harvested from WT mice (*n* = 6) and SOM^−/−^ (*n* = 6); the asterisk (∗) indicates that the cyst numbers were different (*P* < 0.05).

To examine the contribution of somatostatin to parasite burden in cysticercosis, we infected WT mice and somatostatin knockout (SOM^−/−^) mice intraperitoneally with 10 cysts of the ORF strain of* T. crassiceps*, as described [16, 17]. Three months following infection, mice were sacrificed, their peritoneal cavity was opened via laparotomy, and the peritoneal contents were harvested by washing with HBSS (Figure 1). The washings were placed into a Petri dish and the cysts enumerated. The number of cysts in the peritoneum of SOM^−/−^ mice (714 ± 79) was reduced by 83% compared to WT mice (4,222 ± 173; *P* < 0.05).

**Figure 2:**
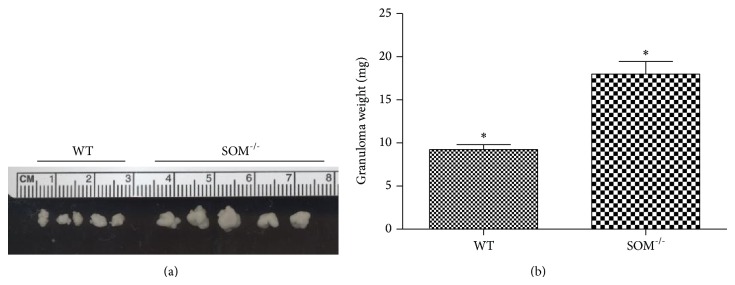
Effect of somatostatin deficiency on size of* T. crassiceps*-induced granulomas. (a) Photograph of representative granulomas removed from infected WT and SOM^−/−^ mice. (b) Weights of granulomas (mean ± SD) obtained from the peritoneal cavity of infected wild type (*n* = 6) and SOM^−/−^ mice (*n* = 6); the asterisk (∗) indicates that the granuloma sizes were different (*P* < 0.05).

To examine the contribution of somatostatin to granuloma formation in cysticercosis, we determined the number and size of granulomas within the peritoneal cavity of* T. crassiceps-*infected WT mice and SOM^−/−^ mice. While there were no differences in the number of granulomas, the size of granuloma determined from their weight (Figure 2) was increased by 95% in SOM^−/−^ mice (17.9 ± 0.6 mg) compared to granulomas obtained from WT mice (9.2 ± 0.2 mg; *P* < 0.05).

### 3.2. Effect of Somatostatin Deletion on Cytokine Levels in* T. crassiceps*-Induced Granulomas

**Figure 3:**
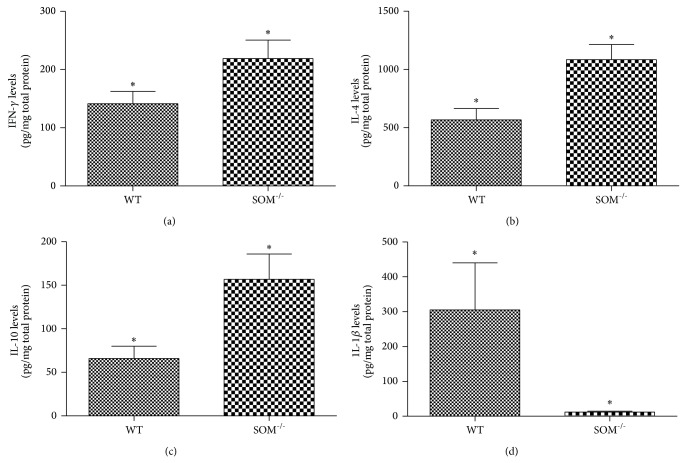
Effect of somatostatin deficiency on levels of cytokines in* T. crassiceps*-induced granulomas. Levels of IFN-*γ* (a), IL-4 (b), IL-10 (c), and IL-1*β* (d) normalized to total protein within peritoneal granulomas obtained from* Taenia crassiceps*-infected WT mice (*n* = 6–8) or SOM^−/−^ mice (*n* = 6–8). Data presented are mean ± SEM; the asterisk (∗) indicates that the cytokine levels were different (*P* < 0.05).

Since Th1 cytokines, IFN-*γ* and IL-2, are known to contribute to granuloma formation, we examined their levels within granulomas from the two groups of mice. Levels of IFN-*γ* in the granulomas derived from SOM^−/−^ mice (219 ± 32 pg/mg total protein; Figure 3(a)) were increased 1.5-fold relative to levels in granulomas of WT mice (142 ± 21 pg/mg; *P* < 0.05); however, levels of IL-2 in granulomas of SOM^−/−^ mice (548 ± 91 pg/mg total protein) were not different from levels in granulomas of WT mice (714 ± 255 pg/mg; *P* > 0.05). These results suggest that IFN-*γ* but not IL-2 is contributing to increased granuloma size in SOM^−/−^ mice.

Since Th2 cytokines, IL-4 and IL-10, are known to downmodulate granuloma formations, we also measured their levels in granulomas from the two groups of mice. Somewhat surprisingly, levels of IL-4 in granulomas from SOM^−/−^ mice (1,084 ± 130 pg/mg total protein; Figure 3(b)) were increased compared to levels in granulomas from WT mice (568 ± 97 pg/mg; *P* < 0.05). The same was true for IL-10 (Figure 3(c)); levels of IL-10 in the granulomas from SOM^−/−^ mice (157 ± 29 pg/mg total protein) were increased compared to levels in granulomas from WT mice (66 ± 15 pg/mg; *P* < 0.05). Thus, the increased granulomatous response seen in SOM^−/−^ mice was not due to reduced levels of the Th2 cytokines, IL-4 and IL-10.

Since the proinflammatory cytokines, IL-1*β*, IL-6, and TNF-*α*, contribute to granulomatous inflammation in tuberculosis and other granulomatous diseases, we also examined their levels in granulomas from the two groups of mice. Surprisingly, IL-1*β* levels in the granulomas derived from SOM^−/−^ mice (12 ± 3 pg/mg total protein; Figure 3(d)) were markedly decreased compared to levels in granuloma from WT mice (305 ± 135 pg/mg; *P* < 0.05); there were no differences in either IL-6 levels (82 ± 22 pg/mg total protein) or TNF-*α* levels (365 ± 48 pg/mg total protein) in granulomas derived from the SOM^−/−^ mice compared to WT mice (189 ± 76 pg/mg and 502 ± 117, resp.; *P* > 0.05 for both). Thus, it is unlikely that increase in these proinflammatory cytokines contributed to the increase in granuloma size observed in SOM^−/−^ mice.

## 4. Discussion

In the current studies, we addressed the role of somatostatin in granulomatous inflammation associated with cysticercosis using a murine model of* Taenia crassiceps* infection [13–16]. In cysticercosis, it is thought that granulomas are formed in response to dying parasites to hasten their elimination from the tissues; once the parasite debri is cleared from the tissue, the granuloma resolves. The molecules responsible for downmodulation of granulomatous inflammation after parasite elimination are not known. Based on our earlier studies showing somatostatin detection in more mature granulomas [18] as well as other studies in schistosomiasis showing that somatostatin plays an immunomodulatory role, we hypothesized that somatostatin may be responsible for resolution of granulomatous inflammation in cysticercosis. Our finding of increased granuloma size in SOM^−/−^ mice supports this hypothesis.

Granulomatous inflammation, while being beneficial through removal of the cyst remnants, clearly can be detrimental causing seizures when inflammation occurs in brain parenchyma. Our findings of decreased cyst burden accompanying increased granulomatous inflammation in SOM^−/−^ mice, however, indicate that there is an additional benefit of granulomatous inflammation, that is, controlling the infection within the peritoneum of infected mice.

We demonstrated that levels of IFN-*γ* were increased in granulomas from infected SOM^−/−^ mice compared to WT mice. Previous studies of* T. crassiceps* infection demonstrated that mice receiving IFN-*γ* had lower parasite levels, while mice receiving anti-IFN-*γ* antibody had larger parasite burdens [21]. Also, IFN-*γ* has been demonstrated to contribute to decreased burden of another parasite,* Toxoplasma gondii*. Pretreatment with IFN-*γ* resulted in a 65% reduction of growth of the parasite within astrocytes [23, 24]. Thus, the increased levels of IFN-*γ* within granulomas may have contributed to the decreased cyst numbers observed in* T. crassiceps*-infected SOM^−/−^ mice.

Based on our findings, another cytokine that may be involved in reduction of parasite burden in* T. crassiceps*-infected SOM^−/−^ mice is IL-1*β*. Studies have shown that IL-1*β* stimulates the growth of* Toxoplasma gondii* in astrocytes [25]. We demonstrated that the level of IL-1*β* was significantly reduced in granulomas from infected SOM^−/−^ mice which correlated with reduced parasite burden. We hypothesize that the same effect observed in* T. gondii* infection may be occurring in* T. crassiceps* infection; IL-1*β* may stimulate growth of cysts in the peritoneum of infected WT mice and its reduction in the granulomas of SOM^−/−^ mice impairs cyst growth in the peritoneum of these animals.

The finding of reduced IL-1*β* in the absence of somatostatin is not completely unexpected. Earlier studies examining the ability of somatostatin to modulate production of IL-1*β*, along with TNF-*α* and/or IL-6, are conflicting. Some studies showed that somatostatin stimulates the production of IL-1*β*, TNF-*α*, and/or IL-6 by human blood cells [26, 27], as well as the expression of IL-1*β* in articular tissues of rats with ongoing adjuvant-induced arthritis [28]. However, other studies showed that somatostatin decreased secretion of one or more of these cytokines [7, 9, 28–31].

The Th2 cytokine, IL-10, has been shown in earlier studies to induce a significant increase in parasite load [21]. Therefore, our results showing increased IL-10 in the somatostatin knockout mice in which the parasite burden is lower were unexpected. We speculate that the effects of IFN-*γ* and IL-1*β* on inhibition of parasite burden likely outweigh the possible stimulatory effects of IL-10.

Our findings have potential implications for treatment and prevention of the detrimental effects of granulomatous inflammation induced as a result of antihelminth treatment in patients with neurocysticercosis and viable cysts. Current options for management of patients with viable cysts include antihelminth treatment along with corticosteroid administration aimed at reducing inflammation. However, corticosteroids can have severe side effects and cannot be used in patients with concurrent latent tuberculosis, strongyloidiasis, and optical cysticercosis. Our finding that somatostatin downmodulates inflammatory responses suggests the possibility of using somatostatin analogues, instead of corticosteroids, as an immunomodulator in these patients to downmodulate granulomatous inflammation in neurocysticercosis.
